# Chronic Aspiration as a Cause for Dendriform Pulmonary Ossifications

**DOI:** 10.5334/jbsr.3603

**Published:** 2024-06-03

**Authors:** Joris Schollaert, Wim Geyskens, Naïm Jerjir

**Affiliations:** 1 VITAZ General Hospital, Sint-Niklaas, Belgium; 2 VITAZ General Hospital, Sint-Niklaas, Belgium; 3 VITAZ General Hospital, Sint-Niklaas, Belgium

**Keywords:** Dendriform pulmonary ossifications, DPO, Computed tomography (CT), Interstitial lung disease, Chronic aspiration

## Abstract

*Teaching point:* Dendriform pulmonary ossifications (DPO) are a rare form of diffuse pulmonary ossifications, in which these ossifications are organised in dendrite-like lines in the periphery of the bases of the lung, most commonly attributed to underlying interstitial lung disease (ILD), but can also be found in patients with chronic aspiration if no other CT findings of ILD are present.

## Case Presentation

A 76-year-old male underwent a chest computed tomography (CT) to exclude lung metastases of a colorectal carcinoma. Medical history showed a long-lasting, nonproductive cough for several years. He was known with symptomatic gastroesophageal reflux disease for a long time. The CT showed no metastases. However, numerous peripheral pulmonary micronodules with high attenuation, best seen on the mediastinal window, were present in both lung bases. These calcified lower lobe nodules were bilateral but asymmetrical and resembled a contiguous cluster of nodules in a branching pattern, that is, dendrite-like. MIP reconstructions (Maximum Intensity Projection) more clearly illustrated the branching pattern of these nodules. The diagnosis of ‘*dendriform pulmonary ossifications’* was made. There was no honeycombing, traction bronchiectasis, or other signs of fibrotic interstitial lung disease (ILD). A large mixed-type hiatal hernia of the stomach was reported.

## Discussion

‘*Dendriform pulmonary ossification’* is a rare disease characterized by micronodules in the peripheral interstitium of the lung, frequently with high attenuation on the mediastinal window (though not necessarily), organized in a branching dendrite-like pattern. In literature, diffuse pulmonary ossifications have been classified histologically in dendriform pulmonary ossification (DPO) alongside the more frequent nodular subtype. The nodular subtype, characterized by lobulated bony micronodules in the alveolar spaces, has been described in patients with passive congestion and other cardiac pathologies (e.g., mitral valve stenosis, chronic left ventricular heart failure . . .).

Typically, the dendriform subtype is found in patients with ILD, most commonly in usual interstitial pneumonia (UIP). These cases are typically accompanied with profound honeycombing and traction bronchiectasis. However, in this case, there were no signs of UIP. Recently, it has been suggested by Gruden et al. that DPO can be caused by chronic aspiration of gastric acid in the absence of UIP [1]. Due to the large mixed-type hiatal hernia and the absence of UIP both on CT and clinically, this hypothesis is also applicable to this case. Increased acidity is postulated to promote the differentiation of fibroblasts and possibly macrophages into osteoblasts, resulting in the micronodules seen on CT.

**Figure:**
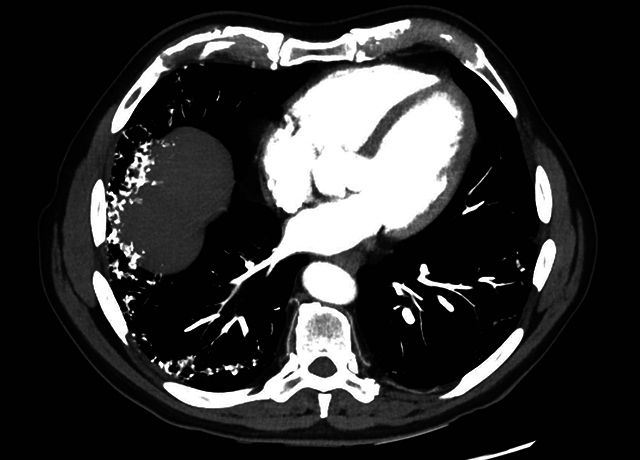


**Figure:**
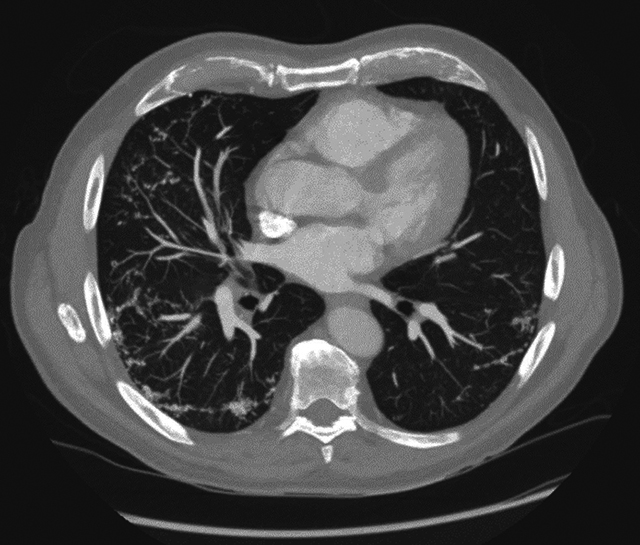


**Figure:**
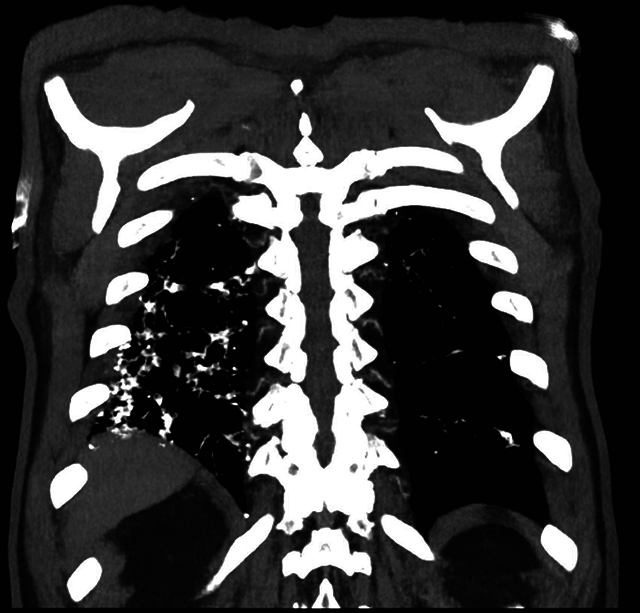


Most commonly, patients with DPO are asymptomatic and are often diagnosed in a postmortem examination. Therefore, on CT, DPO is almost always an incidental finding. They might exhibit a mild cough or dyspnea; however, in most cases, this is due to the pre-existing lung condition (such as chronic obstructive pulmonary disease [COPD] or ILDs such as UIP or nonspecific interstitial pneumonia [NSIP]).
